# Six-month survival of patients with lung cancer admitted to a medical ICU: a retrospective study

**DOI:** 10.1186/cc11017

**Published:** 2012-03-20

**Authors:** O Keller, GL Laplatte, H Lessire

**Affiliations:** 1 CH Pasteur, Colmar, France

## Introduction

ICU admission of patients with lung cancer remains debated because of the poor short-term prognosis. We evaluated the duration of survival of patients admitted to our ICU and looked for factors associated with better survival.

## Methods

All patients with nonresectable lung cancer admitted to our ICU between 1 January 2008 and 31 December 2010 were included in a retrospective study. Postoperative patients were not included.

## Results

Twenty-two patients were included. Seventeen had nonsmallcell lung cancer (NSCLC). One had small cell lung cancer. Fifteen patients (65%) had metastatic disease. Twelve patients were in palliative therapy. The reason for ICU admission was acute respiratory failure in 12 patients (55%), hemorrhage in five patients (23%). Nine patients (41%) had an infection. Fourteen patients (64%) needed invasive mechanical ventilation. One-month survival was 45% (10/22). Six-month survival was 13% (3/22). One-year survival was 0%. One-month survivors showed a nonsignificant trend to lower performance status and severity of disease. All 6-month survivors had metastatic disease. Six-month survivors had nonsignificantly lower performance status (1.7 ± 0.6 vs. 2.7 ± 1.2; *P = *NS). IGS II, SOFA score and duration of mechanical ventilation were significantly shorter in survivors (see Table 1).

**Table 1:** 

	IGS	SOFA	Number of ventilation days
Nonsurvivors	53.2 ± 6.5	5.6 ± 3.8	6.1 ± 6.4
6-month survivors	36.3 ± 9.8	1.3 ± 2.3	0.7 ± 1.2
	*P *< 0.05	*P *< 0.05	*P *< 0.01

## Conclusion

Prognosis of patients with nonresectable lung cancer admitted to the ICU was poor. Metastatic disease did not influence survival in our survey. Patients admitted for a critical illness requiring more than a few days of mechanical ventilation were very unlikely to survive over 6 months.
